# Key Role for CRB2 in the Maintenance of Apicobasal Polarity in Retinal Pigment Epithelial Cells

**DOI:** 10.3389/fcell.2021.701853

**Published:** 2021-06-28

**Authors:** Antonio E. Paniagua, Alicia Segurado, Jorge F. Dolón, Julián Esteve-Rudd, Almudena Velasco, David S. Williams, Concepción Lillo

**Affiliations:** ^1^Institute of Neurosciences of Castilla y León, IBSAL, Cell Biology and Pathology, University of Salamanca, Salamanca, Spain; ^2^Stein Eye Institute and Department of Ophthalmology, David Geffen School of Medicine at UCLA, Los Angeles, CA, United States

**Keywords:** CRB2, cell polarity, epithelium, differentiation, retina, RPE-retinal pigment epithelium, Crb complex, cell junctions

## Abstract

Apicobasal polarity is essential for epithelial cell function, yet the roles of different proteins in its completion is not fully understood. Here, we have studied the role of the polarity protein, CRB2, in human retinal pigment epithelial (RPE) cells during polarization *in vitro*, and in mature murine RPE cells *in vivo*. After establishing a simplified protocol for the culture of human fetal RPE cells, we studied the temporal sequence of the expression and localization of polarity and cell junction proteins during polarization in these epithelial cells. We found that CRB2 plays a key role in tight junction maintenance as well as in cell cycle arrest. In addition, our studies *in vivo* show that the knockdown of CRB2 in the RPE affects to the distribution of different apical polarity proteins and results in perturbed retinal homeostasis, manifested by the invasion of activated microglial cells into the subretinal space. Together our results demonstrate that CRB2 is a key protein for the development and maintenance of a polarized epithelium.

## Introduction

Cell polarity is a key feature of many types of cells, and consists of an asymmetrical distribution of molecules, organelles, and structures within the cell. Epithelial cells exhibit a special category of polarity, named apicobasal polarity, in which distinct apical and basolateral surfaces are exposed to different environments (Rodriguez-Boulan and Macara, 2014). This organization is essential to the barrier function of an epithelium. Apicobasal polarity is established during the differentiation process by the activity of three different protein complexes, the Par complex, the Scribble (Scrib) complex, and the Crumbs (Crb) complex (Johnson and Wodarz, 2003; Ellenbroek et al., 2012). In mammals, the Par complex is comprised of the PAR3, PAR6, and aPKC proteins. It is the first to be established at the plasma membrane and initiates the polarization process by determining the apical domain of the cell. This event is followed by specification of the basolateral surface by the Scrib complex, consisting of SCRIB, DLG, and LGL proteins. Finally, the Crb complex, comprised of the CRB proteins and the PALS1 and PATJ proteins, is recruited more apically than the Par complex, and antagonizes Scrib function to establish apicobasal polarity (Assemat et al., 2008).

One of the most relevant processes that takes place during epithelial polarization is the establishment of the apical junctional complex (Rodriguez-Boulan and Macara, 2014). In vertebrates, adherens and tight junctions form intercellular junctions, thus effecting adhesion of neighboring cells and the barrier function of the epithelium. During formation of the junctional complex, the apical polarity complexes, Par and Crb, integrate into these cell junctions. Coincident with cell junction formation and apicobasal polarization, epithelial cells proliferate to reach an optimal cell density, and then, progressively cease cell division to control tissue size (McCaffrey and Macara, 2011). The importance of cell junction formation during epithelial differentiation (Matter et al., 2005) is manifested by the link between defects in several tight junction proteins and the appearance of tumors, due to defects in the control of the cell cycle (McCaffrey and Macara, 2011; Ellenbroek et al., 2012; Martin-Belmonte and Perez-Moreno, 2012).

Three CRB proteins, CRB1-3, are expressed in mammals. Mutations in the *CRB* genes are the cause of a variety of developmental disorders (Bazellieres et al., 2009). Mutations in *CRB1* and *CRB2* are implicated in retinal dystrophies in humans and deletion of the *Crb2* gene in the mouse retina was reported to perturb development of the photoreceptor layer defects (Den Hollander et al., 1999; Alves et al., 2013; Chen et al., 2019). CRB3 is the most studied CRB protein; it regulates tight junction formation and epithelial cell polarization (Roh et al., 2003; Lemmers et al., 2004; Fogg et al., 2005). Additionally, it is known that both CRB2 and CRB3 are essential for mouse embryonic development (Xiao et al., 2011; Whiteman et al., 2014; Charrier et al., 2015).

The retinal pigment epithelium (RPE) is a monolayer of epithelial cells that is essential for visual function and retinal homeostasis through its support of the photoreceptor cells. The RPE is involved in the turnover of the photoreceptor outer segments, regeneration of the visual pigment, secretion of growth factors, and the transepithelial transport of molecules (Lakkaraju et al., 2020). Fundamental to these roles is the apicobasal polarity of the RPE cells. The RPE expresses components of the three polarity protein complexes (Van De Pavert et al., 2004; Nguyen et al., 2005; Luo et al., 2006; Cui et al., 2007; Park et al., 2011). However, despite the significant role that polarity plays in the RPE, very little is known about how these polarity protein complexes function in this cell type.

Previously, we detected the presence of CRB2 in the RPE (Paniagua et al., 2015). Recently Chen et al. (2019) showed that a missense mutation in *CRB2* causes Retinitis Pigmentosa (RP) in humans. Furthermore, they observed that the mutant protein, although less stable than the wildtype CRB2, is able to trigger epithelial to mesenchymal transition (EMT) when expressed in healthy RPE cells, thus affecting normal RPE function and homeostasis.

Here, we investigated the RPE function of CRB2 during epithelial polarization. Using a combination of cell culture and *in vivo* studies, we demonstrate that CRB2 plays a critical role in the formation and maintenance of the junctional complex.

## Materials and Methods

### Plasmid and Lentiviral Particles Generation

The following shRNAs sequences were cloned into the pLVTHM vector (Addgene): shCONTROL (*E. gracilis* chloroplast gene): 5′-GCGCGCTTTGTAGGATTCG-3′, shCRB2 (*M. musculus CRB2*) 5′-CGAAGTGGATGAGGACGAA-3′, shCRB2#1 (*H. sapiens CRB2*) 5′-CGCAATGACACCAAGGAAA-3′, shCRB2#2 (*H. sapiens CRB2*) 5′- GTGGATCTGTGGACTCATT-3′. 20 μg of pLVTHM containing the specific shRNA, together with 15 μg of psPAX2 and 6 μg of pMD.2G, were co-transfected into confluent HEK293FT cells following the calcium phosphate precipitation assay (Wiznerowicz and Trono, 2003). The culture medium (DMEM supplemented with 10% heat-inactivated fetal bovine serum, 1% MEM Non-Essential Amino Acids, 1% penicillin/streptomycin and 1% sodium pyruvate) was replaced 8 h after transfection, and 48 h later the supernatant containing the lentiviruses was collected. The supernatant was centrifuged at 4700 × *g* for 10 min, filtered using a PVDF membrane with a 0.22 μm pore size, and stored at −80°C. The supernatants destined for subretinal injections were ultracentrifuge at 121,500 × *g* for 2 h at 4°C, dissolved in PBS and stored at −80°C.

### Hf-RPE Cell Culture and Infection

Clonetics Human fetal RPE cells (LONZA) at the fourth passage were seeded at 50000 cells/cm^2^ in Transwell polyester membrane inserts with a diameter of 6.5 mm and a 0.4 μm pore size (Costar) and maintained in Retinal Pigment Epithelial Cell Basal Medium, RtEBM (LONZA), supplemented with L-Glutamine (4 mM), bFGF (5 ng/ml), Gentamicin (30 μg/ml), and Amphotericin (15 ng/ml). For the first three days, 2% fetal bovine serum (FBS) was also included. The medium was replaced every 3–4 days. For silencing experiments, 16,500 Hf-RPE cells were infected with 20 μl of the supernatant containing the lentiviruses on the 1 day of culture, and then removed 3 days later.

### Animal Handling and Subretinal Injections

For the analyses of the CRB2 role in the RPE, postnatal 21-day C57BL/6J mice were anesthetized with 2.0 isoflurane (Forane) inhalation, and the pupils were dilated with a solution containing 1% (w/v) atropine sulfate and 2.5% phenylephrine. One drop of the local anesthetic of 0.5% proparacaine hydrochloride was also administered to the eye. After three minutes, a sclerotomy of the temporal limbus was performed with a 30-G needle, and 1 μl of the supernatant containing the lentiviruses was injected at the subretinal space at a speed of 150 nl/s with a 33-G needle attached to a NanoFil 100 μL syringe and controlled by an UltraMicroPump III system (World Precision Instruments). The transduction was performed using a VSV-G pseudotyped vector that has a higher tropism (almost exclusive) for the RPE cells when injected subretinally (Balaggan and Ali, 2012). Sixty days later the mice were euthanized with carbon dioxide prior to eyes extraction.

For the analyses of CRB2 expression in epithelial tissues, anaesthetized animals were perfused transcardially with a solution containing 4% paraformaldehyde in 0.1 M phosphate buffer at pH 7.4 (PB) and different tissues were dissected out and post-fixed by immersion for 2 h at room temperature in the same fixative. Later, tissues were cryoprotected with a 30% sucrose solution in 0.1 M PB at pH 7.4, embedded in OCT (Tissue-Tek O.C.T.) and 12 μm sections were obtained with a cryostat (Microm HM550 Thermo Fisher Scientific, Waltham, MA, United States).

### Transepithelial Electrical Resistance (TER)

The TER of the Hf-RPE cells in culture, expressed in Ω⋅cm^2^, was measured with an EVOM2 voltohmmeter and STX2 electrodes (World Precision Instruments) that were submerged into each camera of the Transwell, and calculated by subtracting the background of a blank and multiplying the resulting measurement by the surface area of the filter where the cells were cultured.

### Calcium Switch Assay (CSA)

In order to produce an acute depletion of Ca^2+^ at 21 (days in culture) DIC, the standard medium used to culture the Hf-RPE cells was removed and cells were washed three times with HEPES buffer (Sigma-Aldrich). Then, standard medium containing 4 mM of the Ca^2+^ chelating agent EGTA was added to the cells and maintained for 1 h. After that, cells were washed three times with HEPES buffer (Sigma-Aldrich) and Ca^2+^ containing medium was replaced to the culture. The cell junctions’ condition was checked at 24 and 48 h after Ca^2+^ replacement.

### Protein Extraction and Western Blot Analyses

The Hf-RPE cells were lysed in RIPA buffer (150 mM sodium chloride 1.0% Triton X-100, 0.5% sodium deoxycholate, 0.1% sodium dodecyl sulphate, 50 mM Tris, pH 8.0) with a protease inhibitor cocktail (1:1000, Sigma-Aldrich) and Laemmli buffer (2% SDS, 10% glycerol, 700 mM β-mercaptoethanol, 62.5 mM Tris–HCl, pH 6.8, and 0.05% bromophenol blue), and loaded onto a SDS-polyacrylamide gel under reducing conditions. After electrophoresis, the proteins were transferred onto a PVDF membrane, blocked for 1 h at RT in a Tris-buffered saline-Tween (0.1%) (TBST) solution with 2% bovine serum albumin (BSA), and immunolabeled overnight at 4°C with the primary antibodies indicated in Supplemental Experimental Procedures (Table 1), in a TBST solution with 2% BSA. After three washes with the TBST solution, the membranes were incubated with 1:10000 anti-rabbit, mouse or goat IgGs conjugated with horseradish peroxidase (Jackson ImmunoResearch) in 2% BSA and 2% milk in TBST solution for 60 min at RT, washed with the TBST solution, and developed with Clarity ECL Western Blotting Substrate (Bio-Rad), and detected in a chemiluminescent imaging system (MicroChemi 4.2, Berthold Technologies). Densitometry was measured with ImageJ software and minor adjustments to brightness were performed with Adobe Photoshop CS5.

**TABLE 1:** Antibodies and fluorescent molecules used in the study.

**Antibodies and fluorescent molecules**	**Source**	**Catalog number**	**WB**	**IF/IHC**
aPKC	Santa Cruz Biotechnologies	sc-216		1/100
Claudin-19	Abnova	H00149461-M02		1/200
CRB2	Custom-made	REF#23	5 μg/ml	
CRB2	Thermo Fisher scientific	PA5-25628		1/100
Cytokeratin	Sigma Aldrich	C2562		1/100
Phaloidin-FITC	Sigma Aldrich	P5282		1/500
Iba1	Wako Pure Chemical Industries	019-19741		1/750
Ki67	Abcam	ab15580		1/250
Na,K-ATPase	Abcam	ab7671		1/250
Occludin	Thermo Fisher scientific	71-1500	1/200	1/500
PALS1	Abnova	H00064398-A01		1/250
PALS1	Millipore	07-708	1/1000	
PAR3	Millipore	07-330	1/500	1/250
PATJ	Abcam	ab102113		1/200
RPE65	Abcam	ab13826		1/1000
Scribble	Santa Cruz Biotechnologies	sc374139		1/200
ZO-1	Thermo Fisher scientific	33-9100		1/100
β-actin	Sigma Aldrich	A5441	1/5000	
β-catenin	BD Bioscences	610154		1/250
β-catenin	Santa Cruz Biotechnologies	sc-1496	1/100	

*The name of the antigen, the source and catalog number of the antiserum, as well as the concentration and the technique used are indicated in this table. WB: Western-Blot. IF/IHC:Immunofluorescence/immunohistochemistry.*

### Immunofluorescence and Immunohistochemistry Analyses and Imaging

Hf-RPE cells were fixed with 4% paraformaldehyde (PFA) for 10 min at 4°C. For subsequent analysis of changes in the RPE cells using RPE Flat Mounts, after the eyes were isolated, the cornea, lens and retina were removed, the RPE and choroid were fixed by immersion for 10 min in 4% PFA at 4°C. For the RPE Flat Mounts destined to the analyses of the subretinal microglia, these were immersed for 2 h in 4% PFA at 4°C and then the retina was removed. For retina cryosections, the eyes were dissected out and fixed for 2 h in 4% PFA at 4°C. The cornea and lens were then removed, and the eyeballs cryoprotected in a graded sucrose series, into Tissue-Tek O.C.T (Sakura), and then stored at −20°C until sectioning. Tissue sections of 14 μm were obtained with a cryostat (Microm HM560, Thermo Fisher Scientific), and placed onto in Superfrost Ultra Plus (Thermo Fisher Scientific) slides and stored at −20°C until their use.

Following on, cells and tissues were permeabilized with PBS with 0.2% Triton X-100 (PBS-Tx), blocked for 1 h in a solution with 1% BSA and 5% normal serum in PBS-Tx, and then incubated in a solution containing 1% BSA and 2% normal serum in PBS-Tx and the primary antibodies indicated in Table 1 at 4°C. The cells were then washed with PBS-Tx and incubated for 1 h at room temperature with 1:750 Alexa fluor 488 and/or 1:750 Alexa fluor 555 fluorescent secondary antibodies (Life Technologies), and the TOPRO-3 nuclear staining dye (Life Technologies, 1:1000) or DAPI (Sigma-Aldrich, 1:10000) in 1% BSA and 5% normal serum in PBS-Tx. Finally, cells were washed with PBS-Tx and PBS and then the Transwell membranes and Flat Mounts were placed onto a slide with the cells facing the cover slip and mounted using the Prolong Gold antifading reagent (Life Technologies, Carlsbad, CA, United States).

Selected sections were labeled using the Avidin-Biotin Complex (ABC) method. Sections were washed in a PBS solution 0.1 M, pH 7.4 and at last, rinsed in a solution with PBS, methanol and 30% H_2_O_2_ to remove the endogenous peroxidase activity. After rinses in PBS-Tx 0.4 M, pH 8, sections were incubated 72 h at 4°C with CRB2 antibody (Thermo Fisher Scientific 1:100). Following this, sections were washed with PBS-Tx 0.4 M, pH 8, and incubated for 2 h with secondary antibody anti rabbit Ig-G biotinylated (Jackson ImmunoResearch, West Grove, PE, United States) conjugated to horseradish peroxidase in PBS-Tx. Sections were incubated for 3 h in a solution with PBS-Tx 0.4 M, pH 8, and the avidin-biotin peroxidase kit (ABC complex, Vector) and labeling was developed using 3.3’-diaminobenzidine tetrahydrochloride (DAB 0.2%) as chromogen. Negative controls were performed by removing primary or secondary antibodies.

Images were obtained with a light microscope (Axio Observer Z1, Zeiss) with 40×/0.75 and immersion oil 100×/1.30 objectives, or a laser scanning spectral confocal microscope (Leica TCS SP2) with 40×/1.25 and 63×/1.32 immersion oil objectives and with the pinhole set at 1.0 Airy Units. The laser lines of 488, 543, and 633 nm were used to excite the Alexa 488, Alexa 555, and TOPRO3 fluorochromes, respectively, and the three different channels were captured in sequential mode. Epifluorescence images were obtained with a Time-Lapse (Zeiss) microscope with 20×/0.8 and immersion oil 40×/1.3 objectives. Proliferation counting was performed using the ImageJ plugin for Image-based Tool for Counting Nuclei (ITCN) and microglial cells were counted with the ImageJ Cell Counter plugin. Minor contrast and brightness adjustments were performed with Adobe Photoshop CS5 EXTENDED software and Leica Confocal Software.

### Electron Microscopy Analyses

Hf-RPE cells were fixed with 2% PFA and 2% glutaraldehyde in 0,1 M cacodylate buffer, pH 7,4, for 24 h at 4°C, and post-fixed during 2 h in darkness with 1% OsO_4_ (v/v) and 1% K_3_Fe(CN)_6_ (v/v) diluted in ultrapure water. Then, cells were washed with distilled water and dehydrated using a graded ethanol series, and a final step in propylene oxide prior to resin infiltration. Following on, samples were embedded in Epoxy EMbed-812 resin (Electron Microscopy Sciences), ultrathin sections were obtained using an ultramicrotome Ultracut E (Leica), which were contrasted with uranyl acetate and lead citrate and analyzed using a JEOL JEM-1011 HR electron microscope with a CCD Gatan ES1000W camera with iTEM software at the Electron Microscopy Facilities-NUCLEUS of the University of Salamanca. Minor contrast and brightness adjustments were performed with Adobe Photoshop CS5 Extended software and Leica Confocal software. Height and length measurements were carried out with ImageJ software.

### Statistical Analyses

All statistical analyses were performed with SPSS Statistics 20.0 software. Values are expressed as the mean ± standard error of the mean (SEM), Significance differences between groups were evaluated using the parametric Student’s *t*-test or ANOVA, and the Bonferroni’s *post hoc* test, or the non-parametric Mann–Whitney U test or Kruskal-Wallis test. The difference was considered significant when *p* < 0.05 and indicated by ^∗^, and highly significant if *p* < 0.01 and indicated by ^∗∗^. Non-significant differences were indicated by ns.

## Results

### Hf-RPE Cells Differentiate in Culture

To study the condition of the polarity protein complexes during epithelial polarization we developed a simplified protocol for differentiating human fetal RPE (Hf-RPE) cells, as described in the Methods. In particular, we have used a relatively simple, commercially available culture medium; previous studies have used complex media, involving a large number of supplemental components (Hu and Bok, 2001; Maminishkis et al., 2006; Sonoda et al., 2009). Using these culture conditions, we studied the differentiation process by analyzing two of the main features of differentiated epithelial cells: cell quiescence and the establishment of cell to cell junctions (Liao et al., 2010).

**FIGURE 1:**
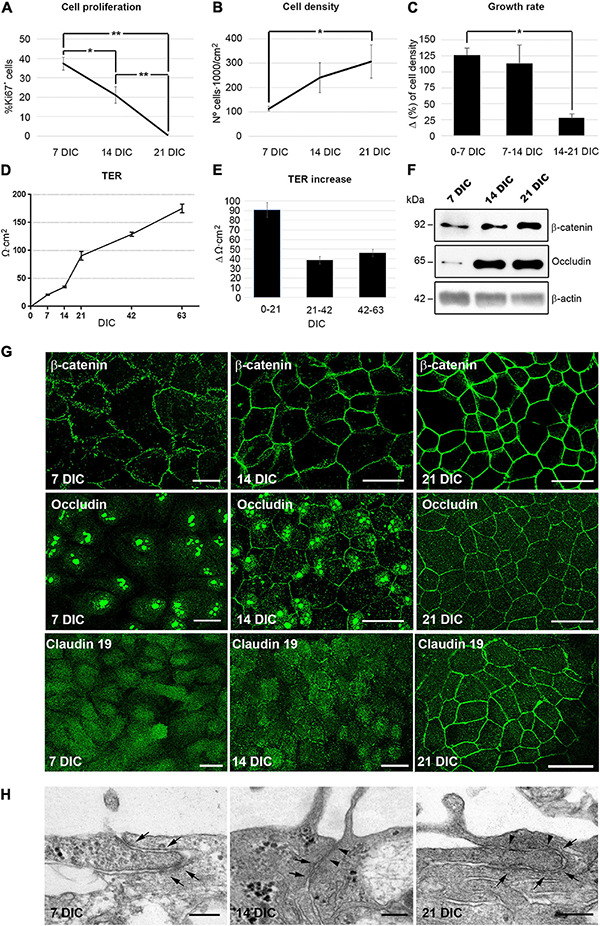
Hf-RPE cells differentiate within 21 DIC. **(A)** Graphical representation of the decrease in the number of proliferating cells from 7 to 21 DIC, **(B)** the increase in cell density from 7 to 21 DIC and **(C)** the decrease in the growth rate expressed as increment (Δ) in cell density within 7 days’ time frames. **(D)** Graphical representation of the highly significant increase of TER in the Hf-RPE cells from 7 to 63 DIC and **(E)** average TER increase within periods of 21 days. **(F)** Western blot analyses showing the expression of adherens junction protein β-catenin and tight junctions protein occludin from 7 DIC onward. β-actin was used as the loading control. **(G)** Confocal microscopy images of β-catenin localization at the plasma membrane from 7 DIC forward, occludin from 14 DIC forward, and claudin-19 from 21 DIC. **(H)** Electron microscopy micrographs showing that adherens junctions are present from 7 DIC onward (arrows), and tight junctions are present from 14 DIC (arrowheads) in Hf-RPE cells in culture. Scale bars in **(G)**: 25 μm, **(H)**: 200 nm. *Significant difference, *p* < 0.05, **highly significant difference, *p* < 0.01.

In order to determine the time point when cell proliferation ceases in culture, and use it as an indicator for quiescence, cells were labeled with antibodies against Ki67, a protein expressed in all phases of the cell cycle except in G0 (Gerdes et al., 1983). In our culture model, the percentage of proliferating cells decreased from 37% ± 3.4 (*n* = 7) at 7 DIC to 21% ± 4.2 (*n* = 6) at 14 DIC as indicated by Ki67 immunolabeling. At 21 DIC, less than 1% [0.6% ± 0.5 (*n* = 4)] of the cells were labeled (Figure 1A and Supplementary Figure 1), indicating that the cell culture had probably reached an optimal cell density and that the contact inhibition pathway had been activated. By quantifying the cell nuclei, we observed that cell density increased until 21 DIC, after which it remained constant at 300,000 cells/cm^2^ (Initial seeding density of 50,000 cells/cm^2^ increased to 113,000 cells/cm^2^ ± 10,000 (*n* = 6) at 7 DIC, 241,000 cells/cm^2^ ± 62,000 (*n* = 5) at 14 DIC, and to 308,000 cells/cm^2^ ± 68,000 (*n* = 3) at 21 DIC; Figures 1B,C and Supplementary Figure 1). Together, all these data indicate that the cell culture reaches an optimal cell density by 21 DIC, when cell proliferation has ceased.

One of the main functions of differentiated epithelia is to minimize the paracellular flow of ions, by establishing adherens and tight junctions at the apicolateral membrane (Rodriguez-Boulan and Macara, 2014). To study the development of this characteristic, the transepithelial electrical resistance (TER) of the cultures was measured with respect to time in culture. The TER increases progressively during the first 21 DIC, reaching 91 Ω⋅cm^2^ ± 8 (*n* = 18). After that, it continues to increase at about half the rate, reaching 175 Ω⋅cm^2^ ± 8 (*n* = 18) after 63 DIC (Figures 1D,E). A TER of approximately 200 Ω⋅cm^2^ has been considered by previous studies to be indicative of a fully differentiated RPE culture, since it is consistent with the physiology of native RPE (Gallemore and Steinberg, 1993; Hu and Bok, 2001; Maminishkis et al., 2006; Sonoda et al., 2009; Hazim et al., 2017).

Next, we analyzed the expression and localization of the adherens junction protein, β-catenin and the tight junction proteins occludin and claudin-19 (Peng et al., 2011). β-catenin was evident at 7 DIC and was localized near the cell periphery, showing an 30% increase in expression level over the following 14 days, when its peripheral localization became more distinct (Figures 1F,G). Occludin expression was very low at 7 DIC but went through a 6-fold increase at 14 and remained constant at 21 DIC (Figure 1F). At 7 DIC, occludin was present in the nuclei, which persisted in some cells at 14 DIC although at this later stage, some staining was also localized to the cell periphery (Figure 1G). Nevertheless, by 21 DIC, occludin was preferentially concentrated at the cell margins (Figure 1G). The nuclear localization of occludin has been found in other cell types, such as astrocytes, where it binds to nuclear proteins, particularly those related to RNA metabolism and nuclear function, performing other roles different to participating in cell junctions (Morgan et al., 2018). Claudin-19 appeared in the cytoplasm at 7 DIC and still only partly localized to the plasma membrane in some cells at 14 DIC (Figure 1G). However, at 21 DIC, this protein was clearly localized to the cell junctions, so that its labeling illustrated the classical epithelial cobblestone organization (Figure 1G). In addition, ultrastructural analysis of the apicolateral membrane of the Hf-RPE cells demonstrated that adherens junctions, but not tight junctions, were formed at 7 DIC (Arrows in Figure 1H). At 14 and 21 DIC, both adherens (Arrows in Figure 1H) and tight junctions (Arrowheads in Figure 1H) were evident.

These results show that, under the described culture conditions, Hf-RPE cells become well-differentiated by 21 DIC. Most cells have ceased proliferating. Cell-cell junctions have formed and paracellular ion flow has decreased (the TER continues to increase after 21 DIC, although at a slower rate).

### Establishment of Apicobasal Polarity During Cell Differentiation

**FIGURE 2:**
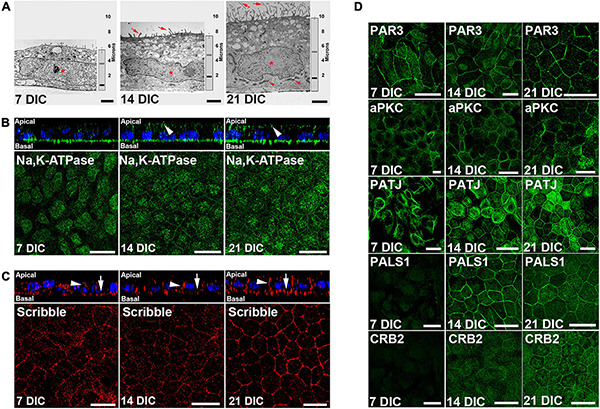
Time course configuration of apical polarity complexes during Hf-RPE cells polarization. **(A)** Electron microscopy images of the RPE cells at 7, 14, and 21 DIC, showing the basal position of the nuclei (asterisk), basal labyrinth and mitochondria (arrowheads), apical microvilli (arrows), as well as the increase in cell height over time (vertical graph bar on the right side of each picture). Vertical bar: medium height of the cells. Gray horizontal bar: relative position of the top of the nucleus. Black horizontal bar: relative position of the bottom of the nucleus. **(B)** Orthogonal view and corresponding maximal projection of a z-stack showing the localization of Na, K-ATPase at the apical membrane from 14 DIC and forward (arrowheads). **(C)** Orthogonal view and corresponding maximal projection of a z-stack showing the localization of Scribble at lateral (arrowheads) and basal (arrows) membranes. **(D)** Confocal microscopy images of Par complex proteins Par3 and aPKC at the plasma membrane of Hf-RPE cells at 7, 14, and 21 DIC. Crb complex protein PATJ is expressed from 7 DIC but localizes at the plasma membrane starting at 14 DIC. Crb complex protein PALS1 is expressed and localized at the cell membrane from 14 DIC and thereafter. Crb complex protein CRB2 is expressed from 14 DIC but localizes at the plasma membrane at 21 DIC. Scale bars in **(A)**: 2 μm; **(B,C)**: 20 μm; **(D)**: 25 μm.

The formation of the circumferential actin belt is an early indicator of polarization in epithelial cells (Miyoshi and Takai, 2008). In our culture model, at 7 DIC, most actin filaments appeared as stress fibers, but at 14 DIC, and especially at 21 DIC, most of the filaments were circumferential (Supplementary Figure 1). The acquisition of apicobasal polarity was also evaluated by transmission electron microscopy of apicobasal sections. Cell height increased from 5.5 μm ± 0.3 (*n* = 15) at 7 DIC, to 7.4 μm ± 0.3 (*n* = 15) at 14 DIC and to 9.9 μm ± 0.2 (*n* = 62) at 21 DIC (Figure 2A). At 7 DIC, the nuclei exhibited fusiform morphology, occupying most of the cytoplasm (Asterisk in Figure 2A). Subsequently, the nucleus was positioned basally, with a flat shape at 14 DIC (Asterisk in Figure 2A), close to the basal labyrinth, while microvilli started to develop at the apical membrane (Arrows in Figure 2A). Then, by 21 DIC, the nucleus acquired a rounded morphology (Asterisk in Figure 2A), fully developed microvilli were present at the apical membrane (Arrows in Figure 2A) and basolateral localization of mitochondria, typical of polarized epithelial cells, was more evident (Arrowheads in Figure 2A).

Morphological changes are accompanied by an asymmetrical distribution of proteins during polarization, so the localization of two such distributed proteins in polarized RPE cells was evaluated. First, we immunolocalized Na,K-ATPase (Figure 2B), a protein that, unlike in other epithelial cells, is localized apically in differentiated RPE cells due to the expression of a β2 subunit specific of this cell type (Hu et al., 1994; Ruiz et al., 1996). This protein was expressed at 7 DIC but only localized to the apical surface from 14 DIC (arrowheads in Figure 2B) indicating the expression of the β2 RPE-specific subunit. We then analyzed the expression and localization of the basal polarity protein, Scribble (Figure 2C), which is also expressed starting at 7 DIC. Its localization at the basal (arrows in Figure 2C) and lateral membranes (arrowheads in Figure 2C) is gradually defined until by 21 DIC, when specifically outlines the basolateral membrane domain (Figure 2C).

Next, we aimed to determine the temporal occurrence of the apical polarity complexes, Par and Crb, in relation to the cell polarization occurring during the first 21 DIC. We investigated the expression and localization of the Par complex proteins PAR3 and aPKC, and the Crb complex proteins PATJ, PALS1, and CRB2. Western blot results showed that PAR3 was expressed at 7 DIC. However, CRB2 and PALS1 expression was not evident until 14 DIC (Supplementary Figure 2). By immunofluorescence, we noticed that PAR3 and aPKC were localized at the cell periphery at all times examined, 7 DIC to 21 DIC (Figure 2D). In contrast, the three Crb complex proteins analyzed exhibited different localization patterns. At 7 DIC, PATJ protein expression was observed, but not that of PALS1 or CRB2 (Figure 2D). At this early time, PATJ was localized to the cytoplasm and formed tangled-like structures (Figure 2D). At 14 DIC, in agreement with Western blot analysis, PALS1 and CRB2 were expressed in the cells, but only PALS1 located at the cell periphery together with PATJ (Figure 2D). At this time, CRB2 and some of the PATJ tangled-like structures remained in the cytoplasm (Figure 2D). Finally, at 21 DIC, CRB2 localized also at the cell periphery, together with PATJ and PALS1 (Figure 2D), completing the Crb complex formation.

Therefore, while the Par complex was established at the cell periphery during an early stage of cell polarization, the Crb complex formation began later and did not appear to be completed until 21 DIC, when CRB2 established a peripheral localization.

### Cytoplasmic and Plasma Membrane Localization of CRB2 in Epithelial Cells *in vivo*

Even though CRB2 is a transmembrane protein that localizes at the plasma membrane where it performs its role as part of the Crb complex, a considerable amount of protein has been reported in the cytoplasm of mouse adult RPE cells (Paniagua et al., 2015). Therefore, as this same pattern was observed in RPE cells *in vitro*, and in order to elucidate whether this is the usual distribution in differentiated epithelial cells, we analyzed its expression and localization in several organs of the adult mouse. Western blot results showed CRB2 expression in kidney, small intestine and testis (Supplementary Figure 2). Immunofluorescence and immunohistochemistry analyses showed that in the kidney, CRB2 is located at the apical plasma membrane of the epithelial cells of the renal tubules, although this expression is not homogenous throughout the tissue, but concentrated in certain regions of the tubules (Supplementary Figure 2). CRB2 is also expressed in epithelial cells of the intestinal villi where most of the cells showed a cytoplasmic localization of CRB2, but some of them also showed a strong labeling at the apical plasma membrane (Supplementary Figure 2). Finally, CRB2 is highly expressed in the tubules of seminiferous epithelium in testis, concentrating at the spermatocytes’ cytoplasm (Supplementary Figure 2).

Therefore, CRB2 is expressed in several epithelial tissues, where it localizes to both the cytoplasm and plasma membrane of epithelial cells.

### Arrival of CRB2 at the Cell Periphery Is Required for Tight Junction Maintenance and Cell Cycle Arrest

The role of CRB2 during differentiation and polarization was investigated by transducing freshly seeded (0 DIC) Hf-RPE cells with lentiviruses containing an shRNA, directed against *CRB2* (shCRB2#1 or shCRB2#2), or a control shRNA (shCTR). The coexpression of GFP with the shRNAs allowed to quantify the efficiency of transfection, that was of 82% ± 1 (*n* = 3) of the overall cell population with each of the 3 shRNAs.

Western blot analysis showed a reduction of a 30.6% in CRB2 protein levels with shCRB2#1 and 47.1% with shCRB2#2 at 14 DIC, when it mainly localizes at the cytoplasm (Supplementary Figure 3). However, reduced CRB2 at 14 DIC did not appear to modify β-catenin, occludin, PAR3 and PALS1 protein levels, and did not affect cell proliferation, cell density or TER (Supplementary Figure 3). Moreover, the localization of β-catenin, PAR3, occludin or PALS1 was not altered in response to reduced CRB2 at 14 DIC (Supplementary Figure 3).

**FIGURE 3:**
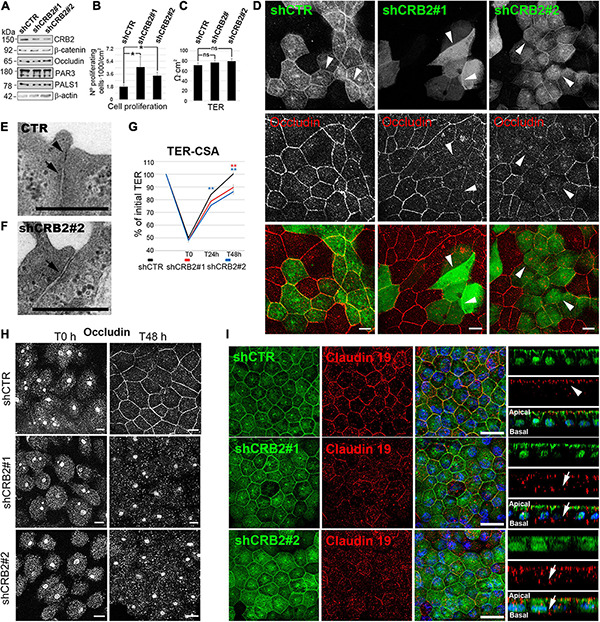
Analyses of the effects of CRB2 knockdown on Hf-RPE cells at 21 DIC. **(A)** Western blot analyses of the CRB2-KD effects at 21 DIC with a reduction of a 59% ± 1.4 (*n* = 3) of CRB2 with shCRB2#1 and 84% ± 2.3 (*n* = 3) with shCRB2#2 showing that the expression levels of the adherens junctions protein β-catenin, the tight junctions protein occludin, the Par complex protein PAR3 and the Crb complex protein PALS1 are not altered. **(B)** Bar graphs showing a significant increase in the number of proliferating cells when CRB2 is silenced with shCRB2#1 or shCRB2#2 compared with the control (shCTR) cells while **(C)** TER remained unaltered at this time point. **(D)** Confocal immunofluorescence images showing that the CRB2 silenced cells with shCRB2#1 and shCRB2#2 show a disruption of occludin labeling at the cell membrane when compared to shCTR infected cells. **(E)** Electron microscope image of a mature cell junction in a control Hf-RPE cell and **(F)** an immature one in a CRB2-KD Hf-RPE cell. **(G)** Line graph of TER development during the CSA of the shCTR, shCRB2#1 and shCRB2#2 cell cultures showing that, from the initial 100% value at 21 DIC, all three experimental models decrease their TER, to the same degree at the first time point measured (T0), 1 h after Calcium is removed from the medium. At 24 h (T24 h) and 48 h (T48 h) after Calcium is re-added to the medium, the CRB2-KD cell cultures recover the TER significantly slower than the control shCTR cell cultures. **(H)** Confocal images of the immunofluorescence labeling of occludin during the CSA of shCTR, shCRB2#1 and shCRB2#2 cells at T0 and T48 h, showing that it is not localized, except in control cells, at the plasma membrane of the CRB2 knockdown. **(I)** Confocal images of immunofluorescence labeling of claudin-19 in the apical membrane (arrows) of cells expressing control shRNA (shCTR) but miss localized to the cytoplasm and basal domain (arrows) in CRB2-KD (shCRB2) cells. Scale bars in **(D,H)**: 10 μm; **(E,F)**: 500 nm; **(I)**: 20 μm. *Significant difference, *p* < 0.05, **highly significant difference, *p* < 0.01.

In contrast, significant changes in some of these features were evident at 21 DIC, when CRB2 also localizes at the cell periphery. CRB2 levels were reduced a 59% with shCRB2#1 and 84% with shCRB2#2 at this time (Figure 3A and Supplementary Figure 3). The number of proliferating cells was increased by 2.5 ± 0.8 fold (*n* = 10) with shCRB2#1 and 1.9 ± 0.3 fold (*n* = 10) with shCRB2#2, when compared with the control shCTR cell cultures (1.0 ± 0.1; *n* = 10) (Figure 3B). Our results showed that CRB2 reduction did not alter the protein levels of β-catenin, occludin, PAR3 and PALS1 at 21 DIC (Figure 3A) and did not affect cell density (data not shown) or TER (Figure 3C). Nevertheless, upon comparing the subcellular localization of cell junction proteins and apical polarity complexes, we found that while β-catenin, PAR3 and PALS1 were localized normally at the cell periphery (Supplementary Figure 3), occludin staining at the plasma membrane was reduced in some CRB2-knockdown (CRB2-KD) cells (Arrowheads in Figure 3D).

Although the TER after 21 DIC showed no significant differences upon CRB2 reduction, the finding that the distribution of a critical tight junction protein was affected, suggested further morphological and functional investigation. Therefore, we first analyzed the cell junctions by conventional electron microscopy and found that, in contrast to control cells, where both tight (Arrowhead in Figure 3E) and adherens (Arrow in Figure 3E) junctions sealed the most apical membrane in 86.7% (*n* = 30) of the cell junctions analyzed, in CRB2-KD cells only 58% (*n* = 50) of the cell junctions presented both components and 42% only show an adherens (Arrow in Figure 3F), but not a clear tight junction structure. Therefore, CRB2-KD cells present some gaps in the belt formed by tight junctions that surrounds every cell. Then, we tested whether the kinetics of tight junction formation was affected in CRB2-KD cells. Following a classical approach for analyzing cell junction formation (Nigam et al., 1992; Hurd et al., 2003), we measured TER recovery after an acute depletion of Ca^2+^. Upon removal of Ca^2+^ from the culture medium for 1 h, the TER of the control and two experimental groups all decreased to ∼50% of the initial values (shCTR: 50.0% ± 1.2; *n* = 56, shCRB2#1: 48.3% ± 1.4; *n* = 55, and shCRB2#2: 48.0% ± 1.4; *n* = 57) (Figure 3G). After return to Ca^2+^-containing culture medium for 24 h, the TER of the shCTR-treated cultures was significantly higher (84.0% ± 1.7; *n* = 46) than that of shCRB2#2 cultures (75.5% ± 1.6; *n* = 47), although not significantly higher than that of shCRB2#1 cultures (78.7% ± 1.7; *n* = 45). After 48 h in Ca^2+^-containing culture medium, the shCTR infected cell cultures had reached 100% ± 1.3 (*n* = 37) of the initial TER, which was significantly higher than that observed in both shCRB2#1 (89.6% ± 2.3, *n* = 37) and shCRB2#2 (86.3% ± 1.9, *n* = 38) cultures (Figure 3G). Therefore, tight junction formation is retarded in the CRB2-KD cell cultures.

In order to investigate whether the reestablishment of cell junctions and polarity complexes were also affected by the reduction of CRB2, we analyzed the localization of β-catenin, occludin, PAR3 and PALS1 by immunofluorescence during this process. In a Calcium Switch Assay (CSA) performed at 21 DIC, after Ca^2+^ depletion, all four proteins, that initially localize at the plasma membrane (Figure 3D and Supplementary Figure 3) were absent from the cell periphery (Figure 3H and Supplementary Figure 3). However, after 48 h of recovery, when these proteins were already localized at the periphery in control cells, only β-catenin and PAR3 were properly localized in CRB2-reduced cells (Supplementary Figure 3). PALS1 showed an irregular distribution, with a sparce staining only in some plasma membrane profiles (Supplementary Figure 3) and occludin staining remained entirely in the cytoplasm (Figure 3H). Therefore, CRB2 knockdown affects the reestablishment of tight junctions at the plasma membrane after Ca^2+^ depletion.

Finally, we investigated claudin-19 localization, a very sensitive marker for well-developed tight junctions specific for human RPE cells and found that it mislocalized to the cytoplasm in CRB2-KD cells (Arrows in Figure 3I) in contrast to the almost exclusive apical staining obtained in cells expressing the control shRNA (Arrowheads in Figure 3I).

We conclude that the arrival of CRB2 at the cell membrane controls the maintenance and reestablishment of the tight junction proteins occludin and claudin-19, and the Crb complex protein PALS1, once these junctions are formed and later disrupted, but not during *de novo* establishment. However, CRB2 does not perform the same role for the adherens junction protein β-catenin and the Par complex protein PAR3.

### *In vivo* CRB2 Knockdown Causes Molecular and Morphological Alterations in Mouse RPE Cells and Subretinal Changes

It has been reported that the changes observed in different types of cells when Crb complex proteins are altered *in vitro* manifest themselves as serious disorders when they occur *in vivo* (Den Hollander et al., 1999; Park et al., 2011; Alves et al., 2013; Whiteman et al., 2014). Hence, we investigated the effect that CRB2 knockdown had on differentiated RPE cells *in vivo*. We injected lentiviral vectors containing GFP and either a previously validated shRNA against mouse *Crb2* mRNA (shCRB2) (Paniagua et al., 2015), or the shRNA control (shCTR), into the subretinal space of 21-day-old mice. The effects of *Crb2* knockdown were analyzed 60 days after the injection.

**FIGURE 4:**
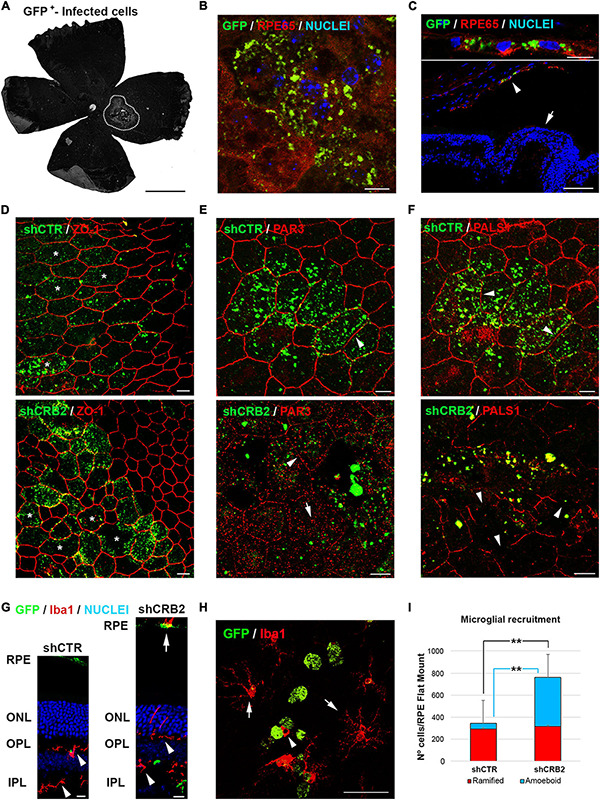
Analyses of the effects of CRB2 knockdown in differentiated RPE cells *in vivo*. **(A)** Representative epifluorescence image of a Flat Mount 60 days after injection with the infection area surrounded by white dots. **(B)** Confocal microscopy image of infected GFP^+^ cells in a Flat Mount where the RPE specific protein RPE65 has been labeled in red and nuclei in blue. **(C)** Confocal microscopy image of infected GFP^+^ cells in a retinal cryosection where RPE65 labeling (red) shows that the infected cells are only RPE cells (arrowhead) and not the neural retina (arrow). **(D)** Confocal microscopy images of RPE cells in a Flat Mount showing that the knockdown of CRB2 does not alter ZO-1 expression or localization but does produce modifications in the morphology of the RPE cells (asterisks). **(E)** Confocal microscopy images of RPE cells in a Flat Mount showing that PAR3 localization changes from a continuous and broad labeling of the plasma membrane in control shCTR cells (arrowhead) to a scarce and scattered labeling of the plasma membrane (arrowhead) and cytoplasm (arrow) in CRB2 knockdown cells. **(F)** Confocal microscopy images of RPE cells in a Flat Mount showing that PALS1 localization changes from a continuous labeling of the plasma membrane in shCTR infected cells (arrowhead) to an interrupted and patchy labeling of the plasma membrane in shCRB2 knockdown cells (arrow). **(G)** Confocal microscopy images of retinal cryosections of the infection area where microglial cells stained with Iba1^+^ are found in both plexiform layers (arrowheads) and the subretinal space (arrow). Nuclei are stained in blue. **(H)** Confocal microscopy images of a Flat Mount where infected GFP^+^ RPE cells (in green) are surrounded by ramified Iba1^+^ (arrows) and amoeboid (arrowhead) microglial cells (in red). **(I)** Bar graph showing that the number of microglial cells in the shCRB2 infected Flat Mounts (761 ± 54; *n* = 6) is significantly higher than in shCTR infected Flat Mounts (343 ± 106; *n* = 6). This increase was, however, not due to the ramified microglial cells, whose number was similar in in both shCTR (294 ± 94; *n* = 6) and shCRB2 (315 ± 56; *n* = 6) infected Flat Mounts but to the amount of amoeboid cells, whose number was significantly higher in shCRB2 (447 ± 146; *n* = 6) compared to shCTR (49 ± 34; *n* = 6) infected Flat Mounts. Scale bars in **(A)**: 1 mm; [**B,C** (bottom), **D–G**]: 10 μm; (**C** (upper), **H**): 50 μm. **Highly significant difference, *p* < 0.01.

The transduced area was determined by identifying GFP-positive cells. It was focused at the site of injection and represented 10% ± 2 (*n* = 15) of the RPE. There was no significant difference in the size of the GFP-positive areas between shCTR and shCRB2 (Figure 4A). The GFP was mostly in the RPE cells, which were identified by immunolabeling with antibodies against RPE65 (Figure 4B and Arrowhead in 4C), and not the neural retina (Arrow in Figure 4C).

CRB2 knockdown did not affect the distribution of the ZO-1 protein (Figure 4D), but the distributions of PAR3 (Figure 4E) and PALS1 (Figure 4F) were perturbed, with a large loss of the peripheral localization of both proteins, included in those that were not transduced, probably because a crosstalk with the affected cells. We then decided to analyze if these changes in the RPE cells affected the retinal condition. One of the main signs of retinal impairment is the invasion of activated microglia into the subretinal space, as in normal conditions this an immunosuppressed environment controlled by the secretion of inhibitory factors by the RPE cells that prevents the entrance of these cells into the subretinal space (Rashid et al., 2019). Therefore, we labeled microglial cells with the specific marker Iba1 in retinal cryosections (Figure 4G). We found that although microglial cells were distributed within both plexiform layers in both shCTR and shCRB2-transduced retinas (Arrowheads in Figure 4G), there was a noticeable higher number of them in the subretinal space (the extracellular region between the apical RPE and the photoreceptor cells) of the CRB2-KD (Arrow in Figure 4G). The presence of microglial cells at the subretinal space is often associated with aging and retinal damage (Li et al., 2015), and it has been shown to promote photoreceptor cell loss (Zhao et al., 2015). Therefore, we quantified the number of microglia cells that had invaded the subretinal space. Both amoeboid phagocytic (Arrowhead in Figure 4H) and ramified non-phagocytic (Arrow in Figure 4H) microglia were identified in RPE Flat mounts. The number of amoeboid phagocytic cells was significantly higher in shCRB2 than in shCTR infected Flat Mounts (Figure 4I). Cells of both phenotypes were distributed along the subretinal space (Supplementary Figure 4), although there was a slightly higher amount of both cell types at the injection site (Yellow circles in Supplementary Figure 4). The arrival of microglia suggests that the retinas will subsequently deteriorate.

## Discussion

Although most of the proteins that make up the Crb complex, namely CRB3, PALS1, and PATJ, are known to play a key role in apicobasal polarity and tight junctions establishment in epithelial cells (Roh et al., 2003; Lemmers et al., 2004; Straight et al., 2004; Shin et al., 2005), no such role had been identified for CRB2. Here, we demonstrate that, in differentiating cultures of retinal pigment epithelial cells, CRB2 functions during a late stage in the formation of the junctional complex and cell cycle arrest. We also show that CRB2 is required for the *in vivo* maintenance of RPE organization in the adult mouse retina.

### Hf-RPE Cell Culture: A Simplified Protocol for Culturing and Studying the Biology of Polarized Retinal Pigment Epithelial Cells

In order to study the role of CRB2 in epithelial cells *in vitro*, we established a new protocol for the culture of human fetal RPE (Hf-RPE) cells. This protocol is based on the use of commercially available Hf-RPE cells and culture media and represents an improvement over past methods due to its relative simplicity. We have analyzed the cell features within the first 21 days of culture when Hf-RPE cells polarize to a level that is similar to other well established human fetal RPE culture protocols (Hu and Bok, 2001; Maminishkis et al., 2006; Sonoda et al., 2009) and to those of epithelial cells *in vivo* (Bonilha, 2014). Simultaneously, these cells progressively differentiate, undergoing cell cycle arrest and the development of physiological epithelial features, such as the formation of a barrier by the formation of a junctional complex (Matter and Balda, 2003; Matter et al., 2005; Rizzolo et al., 2011). The TER continued to rise for at least an additional 6 weeks after 21 DIC, under our culture conditions, indicating that the cells become polarized and quiescent before complete differentiation. Note that extraordinarily high TER measurements have been reported for hfRPE cells maintained in culture for over a month (Ablonczy et al., 2011). In conclusion, our described protocol provides for the culture of epithelial cells that develop mature epithelial phenotype features and can be employed to study the biology of the RPE.

### The Expression and Localization of the Apical Polarity Complexes Are Coincident With the Polarization of the RPE Cells

By the analyses of the expression and location of the apical polarity proteins during the first 21 days of culture, we have described a relationship between the localization of these proteins at the plasma membrane and the polarization and differentiation process.

Initially, Par complex proteins PAR3 and aPKC, and the basolateral protein, Scribble, are expressed and localized at the plasma membrane, when the epithelial cells still show a very low degree of polarity. The adherens, but not tight junctions, are being established, and many of the cells are in a proliferative state. At the same time, the Crb complex protein PATJ, but not PALS1 or CRB2, is also expressed, although it shows a cytoplasmic localization. Hence, Par and Scribble are the first polarity complex that localize to the plasma membrane, where they remain during the rest of the polarization process. This is comparable to cellularization in *Drosophila melanogaster*, where Scribble and the PAR3 homolog, Baz, initiate epithelial polarization and regulate adherens junction formation (Bilder and Perrimon, 2000; Harris and Peifer, 2004, 2005; Coopman and Djiane, 2016). Later, the Crb complex proteins, PALS1 and CRB2, begin to be expressed, but only PALS1, together with PATJ, locate in the plasma membrane and progressively change their localization, while CRB2 remains at the cytoplasm. At the same time, while still mitotically active, the initial establishment of the tight junctions in the RPE cells start to show some degree of apicobasal polarity, since Na,K-ATPase localizes at the apical membrane. Thus, the recruitment of the Crb complex maintains the polarization process, although its establishment at the plasma membrane begins without the contribution of CRB2. Finally, CRB2 completes the formation of the Crb complex by localizing to the plasma membrane at the same time as claudin-19, and Scribble perfectly delineates the basolateral domain. At this time (21 DIC), most of the RPE cells are no longer proliferating and show maximal apical-basal growth. Therefore, this key polarization stage concludes at the same time as CRB2 localizes to the junctional complex.

### CRB2 Knockdown Alters Tight Junction Maintenance in Polarized Epithelia

Recently, a missense mutation of *CRB2* (p.R1249G) has been reported to cause retinitis pigmentosa (Chen et al., 2019). Interestingly, this mutation disturbs the stability of not only the transcribed CRB2-mutant mRNA and the encoded CRB2-mutant protein but of the wild type protein when both are expressed in combination in undifferentiated ARPE-19 cells. The mutant protein also affects to the cell homeostasis and triggers EMT. These findings suggest that a reduction in the levels of CRB2 in RPE cells are more prone to be causing non-syndromic retinal dystrophies in humans than mutations leading to the complete absence of the protein, since the lack of CRB2 during gastrulation causes embryo lethality (Xiao et al., 2011).

Here, we explored the effects of CRB2 knock down during differentiation and in fully polarized human RPE cells *in vitro* as well as in differentiated mouse RPE *in vivo* and observed that CRB2 downregulation is sufficient to cause significant changes in key features of the RPE both *in vitro* and *in vivo*.

One of the main known functions of the Crb complex in epithelial cells is its role in tight junction formation and maintenance (Medina et al., 2002; Bazellieres et al., 2009). Our results confirm this observation, as we have seen that although some tight junctions are able to form in cells with reduced levels of CRB2, the tight junction belt, a structure that completely encircles the control cells, shows some gaps in CRB2-KD cells and it is not reestablished at the plasma membrane at the same speed as in control cells after calcium switch assays. On the other hand, the adherens junction belt seems to be unaltered in cells with low levels of CRB2.

This is in agreement with the observation that, in CRB2-KD cells, occludin, claudin-19, and PALS1, but not β-catenin or PAR3, disassemble from the plasma membrane, even though expression levels in the entire cell culture remain unaltered. It is important to note that the localization of PALS1 is not completely disrupted, so it is possible that some of the proteins of the PALS1 pool still form part of the Crb complex at the plasma membrane, perhaps with another CRB isoform, such as CRB3. Therefore, in our model, CRB2 knockdown affects the Crb protein complex and tight junctions, but not adherens junctions or the Par complex. In fact, recent studies (Tan et al., 2020), by using detailed imaging and proteomics to dissect the spatio-molecular organization of the apical-lateral border in fully polarized MDCK-II cells have resolved the apical junctional complex and uncovered a novel polarity domain (vertebrate marginal zone or VMZ). This VMZ is located apically to the tight junctions and it is defined by the Crb complex, which in this case seems to be led by CRB3. They describe that the expression of PALS1 and PAR3 in this model is crucial for VMZ and tight junction stability. The authors also concluded that VMZ is a common feature of intestinal and renal epithelial cells, so it seems feasible that it may occur in RPE cells as well.

Furthermore, CRB2 knockdown causes a significant increase in cellular proliferation at the time when this protein normally reaches the plasma membrane (21 DIC). However, it does not affect cell density. Apoptotic cells were barely detectable at this time (data not shown), indicating that cell death is not significant. Therefore, a plausible explanation is that the method employed for the quantification is not sensitive enough for detecting differences in such a small number of cells proliferating at 21 DIC (around 0.6% in wild type cells and 1.5% in knockdown-CRB2 cells). Even though the total number of cells proliferating is not high, it could be dangerous if this state is sustained over time *in vivo* since maybe this is why epithelial cells are the origin of most human tumors (McCaffrey and Macara, 2011). Indeed, many of the polarity proteins have been discovered as tumor suppressors or have been related to the onset or malignancy of different types of tumors (Assemat et al., 2008; Karp et al., 2008; Loie et al., 2015; Mao et al., 2015). These results are consistent with studies that described the correlation of defects in CRB2 with an increase in proliferation during the differentiation of neural cells (Boroviak and Rashbass, 2010; Alves et al., 2013), but the present study represents the first report relating CRB2 to this process in epithelial cells. Additionally, in the present work we have found CRB2 to be expressed in diverse epithelial tissues and based on the results obtained after the ablation of CRB2 expression in RPE cells, it would be interesting to better understand its function in those tissues under normal or disease conditions.

Interestingly, all the observed effects of the CRB2 knockdown occur at the time when the protein is normally first localized to the plasma membrane. We cannot discard the possibility that the knockdown achieved at 14 DIC (less than 50% reduction in CRB2 expression with both shRNAs) is not enough to cause any significant effect. However, since the lentiviral transduction efficiency was very similar at 21 DIC (82% ± 1), but the knockdown is much more efficient (84% reduction in CRB2 expression), this result seems to be a basal translation level that may escape from shRNA activity.

What function, then, does CRB2 have when the cell is not fully polarized, and this protein is distributed in the cytoplasm and not being a functional part of the Crb complex at the plasma membrane? It has been reported that the protein p60 AmotL2 retains the CRB3 isoform in the cytoplasm when epithelial cells lose their polarity due to the inhibition of transport by the TRAPPII (Transport Protein Particle II) complex, which regulates the transport of vesicles from the Golgi apparatus to the plasma membrane in polarized cells (Mojallal et al., 2014). The localization of CRB2 in the cytoplasm of differentiating RPE cells as well as other cell types like the ones at the seminiferous tubes may be the result of a similar regulatory process. Therefore, it still needs to be analyzed if the protein performs a specific function at the cytoplasm, or the protein is just trafficking to its final location.

### CRB2 Knockdown Alters RPE Stability *in vivo*

Finally, we have shown that normal levels of CRB2 are necessary for maintaining the stability of differentiated RPE cells *in vivo*. The CRB2 knockdown in the intact mouse RPE causes the displacement of PALS1 and PAR3 from the plasma membrane but does not affect ZO-1 localization. Interestingly, we find PALS1 and PAR3 displacement in cells that do not show shCRB2 transduction, probably because an intercellular crosstalk that causes the reduction of these proteins in non-transduced cells. This phenotype slightly differs from what we found *in vitro*, where TJs and PALS1, but not PAR3, are affected in the CRB2-KD cells. This could be because of the different species used in each approach (human vs. mouse), because of the different environment of each model (mouse eye vs cell culture) or a combination of both. However, these results are consistent with studies showing that the lack of CRB2 and CRB3 leads to the disassembly of polarity proteins within epithelial tissue during mouse embryo development, but it never affects the cell junctions (Xiao et al., 2011; Whiteman et al., 2014; Charrier et al., 2015). Despite these alterations, we have not been able to detect an increase in either apoptosis, or in proliferation (data not shown), as reported when CRB2 and PALS1 are silenced in neural tissues *in vivo* (Kim et al., 2010; Alves et al., 2013) or in undifferentiated ARPE19 cells transfected with *CRB2* (p.R1249G) missense mutation *in vitro* (Chen et al., 2019). These effects might be more frequent when CRB2 is lacking in non-differentiated cells and not in complete polarized epithelial cells, where cell junctions are completely stabilized and confront these mechanisms.

We have not observed any substantial alteration in the neural retina, preceding an increase in activated phagocytic microglial cells in the subretinal space, suggesting that the microglial response is due to perturbations in the RPE. The presence of the microglia, however, suggests that retinal pathogenesis is likely to follow (Li et al., 2015). Hence, it is possible that a sustained knockdown of CRB2 in the RPE eventually leads to RPE pathogenesis, with cell non-autonomous effects resulting in photoreceptor degeneration, a hypothesis also supported by the results obtained by Chen et al. (2019).

## Data Availability Statement

The original contributions presented in the study are included in the manuscript/Supplementary Material, further inquiries can be directed to the corresponding author/s.

## Ethics Statement

The animal study was reviewed and approved by the Bioethics Committee of the University of Salamanca.

## Author Contributions

AP and CL conceived the presented idea. AP, DW, and CL designed the methodology and wrote the original draft. AP, AS, JD, and JE-R performed, validated, and carried out the formal analysis of the experiments. AV, DW, and CL contributed with funding, resources, and supervision. All authors validated the analysis, completed the review and editing of the manuscript, contributed to the final manuscript, and approved the submitted version.

## Conflict of Interest

The authors declare that the research was conducted in the absence of any commercial or financial relationships that could be construed as a potential conflict of interest.
