# Supplementary figures and images for: Key Role for CRB2 in the Maintenance of Apicobasal Polarity in Retinal Pigment Epithelial Cells
Source: Front Cell Dev Biol. 2021 Jun 28;9:701853. doi: 10.3389/fcell.2021.701853 (PMC8273544; doi:10.3389/fcell.2021.701853)

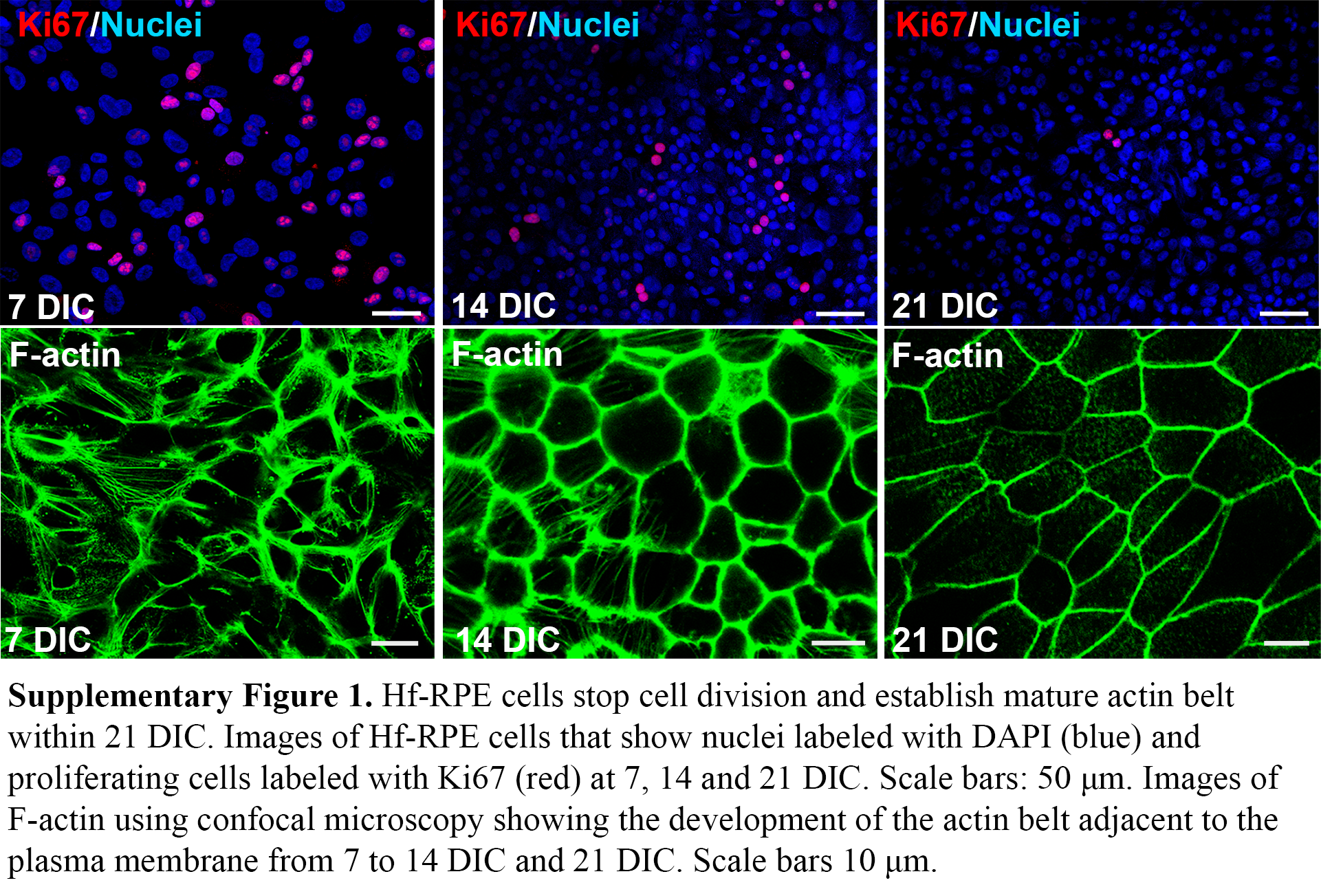

Supplement: Supplementary file 1 [file Image_1.TIF]

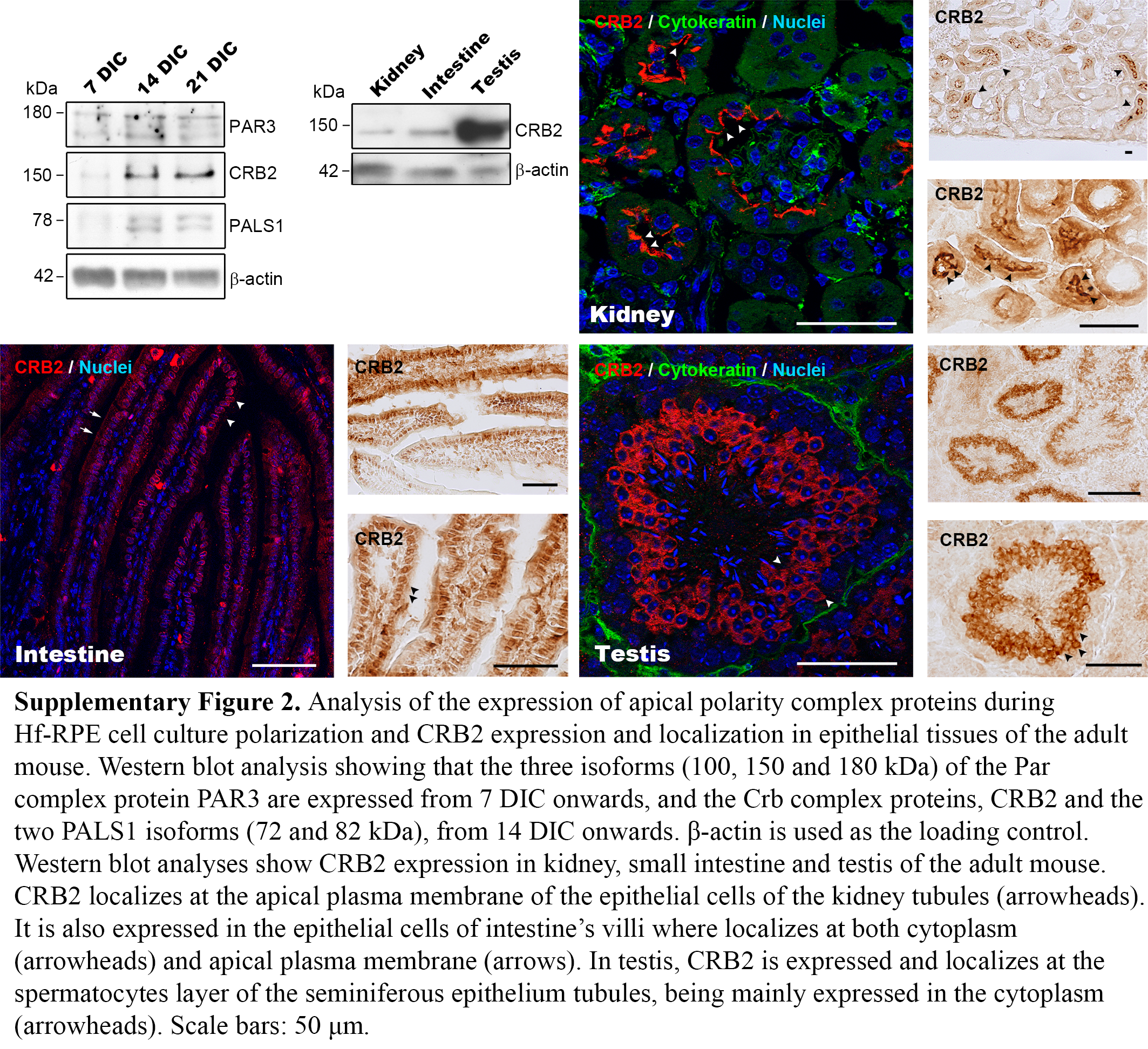

Supplement: Supplementary file 2 [file Image_2.TIF]

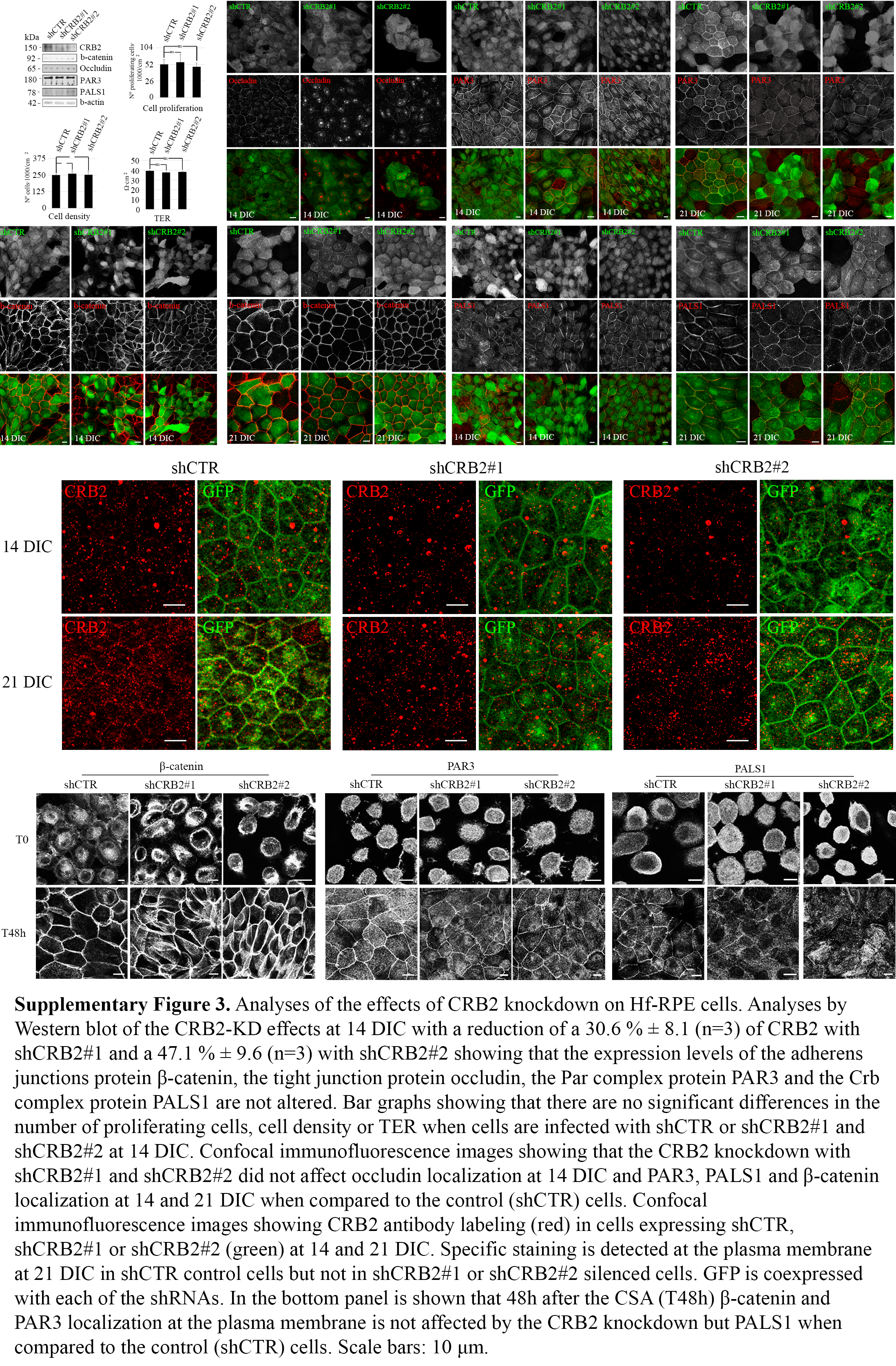

Supplement: Supplementary file 3 [file Image_3.TIF]

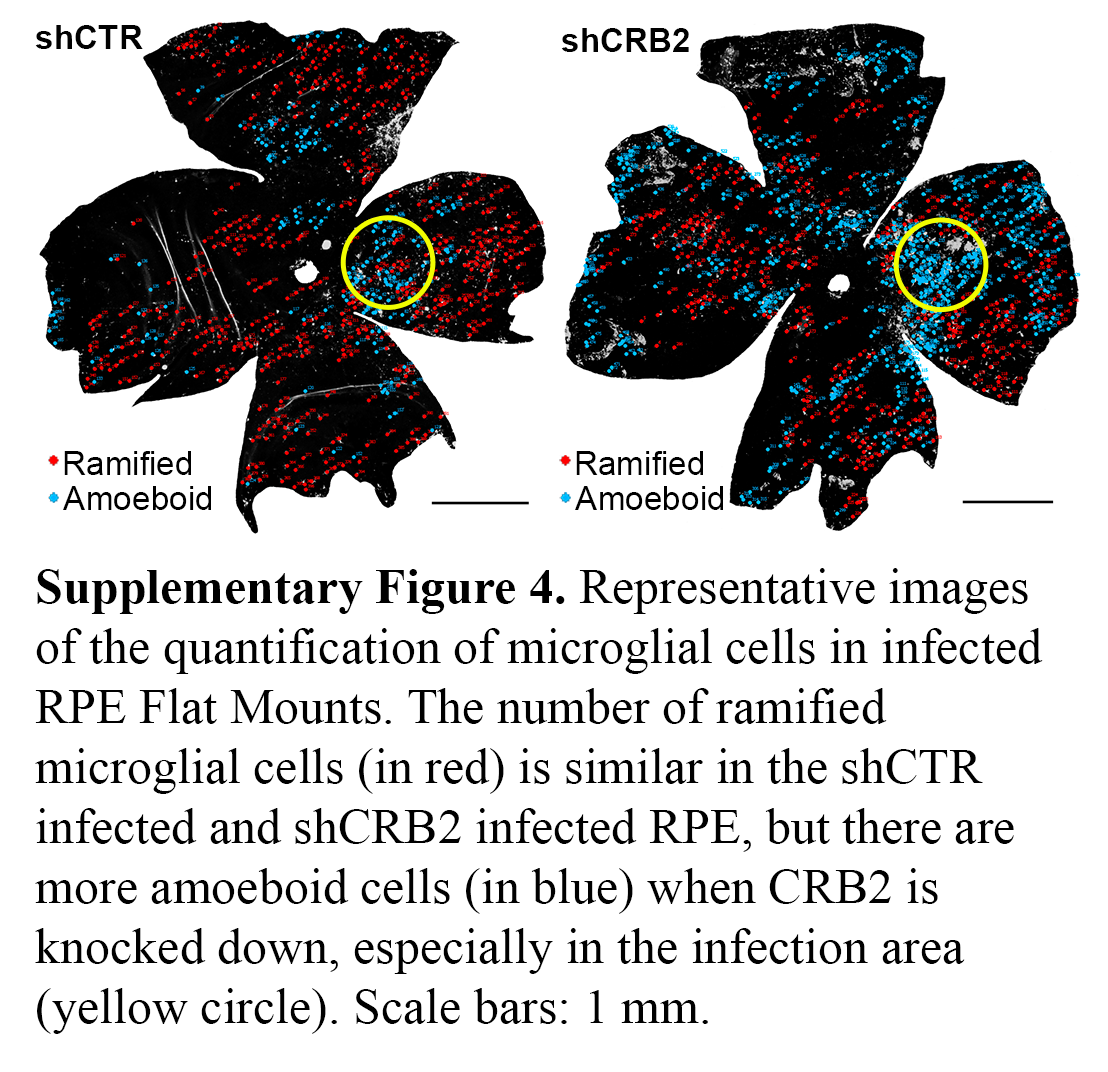

Supplement: Supplementary file 4 [file Image_4.TIF]
